# Regulation of epithelial growth factor receptors by the oncoprotein E5 during the HPV16 differentiation-dependent life cycle

**DOI:** 10.1016/j.tvr.2025.200315

**Published:** 2025-03-07

**Authors:** Mariano A. Molina, Sneha Biswas, Omar Jiménez-Vázquez, Jason M. Bodily

**Affiliations:** a Department of Pathology, Vrije Universiteit Amsterdam, Amsterdam UMC Location VUmc, Amsterdam, the Netherlands; b Cancer Center Amsterdam, Imaging and Biomarkers, Amsterdam, the Netherlands; c Instituto de Ciencias Médicas, Las Tablas, Panama; d MSD Animal Health, Boxmeer, the Netherlands; e Independent Researcher, Utrecht, the Netherlands; f Department of Microbiology and Immunology and Feist-Weiller Cancer Center, Louisiana State University Health Sciences Center, Shreveport, LA, USA

**Keywords:** E5, EGFR, TGFBR, KGFR, FGFR2b, HPV16

## Abstract

Human papillomavirus (HPV) 16 infection initiates upon viral entry into the basal cells of the epithelium. The virus manipulates signaling pathways to complete its life cycle, which depends on cellular differentiation. The virus expresses the oncoproteins E5, E6, and E7 to promote immune evasion, cell cycle progression, apoptosis inhibition, and viral replication. The least studied viral oncoprotein is E5 (16E5), which can regulate epithelial growth factor receptor (GFR) signaling pathways. GFRs such as transforming growth factor-beta receptor (TGFBR), epidermal growth factor receptor (EGFR), and keratinocyte growth factor receptor (KGFR) have essential roles in cell growth, differentiation, and proliferation. These receptors obtain their ligands from the microenvironment, and once activated, regulate cellular behavior in the epithelium. GFRs therefore represent valuable targets for the virus to establish and maintain a cellular environment supportive of infection. The ability of 16E5 to regulate proliferation and differentiation varies through the differentiating epithelium, making it necessary to adequately describe the association between 16E5 and GFRs. Here we summarize the regulation of GFR signaling pathways by 16E5, discuss the roles of stromal growth factors, and outline unresolved questions over cellular differentiation and proliferation during the HPV life cycle.

## 1 Introduction

**Fig. 1:**
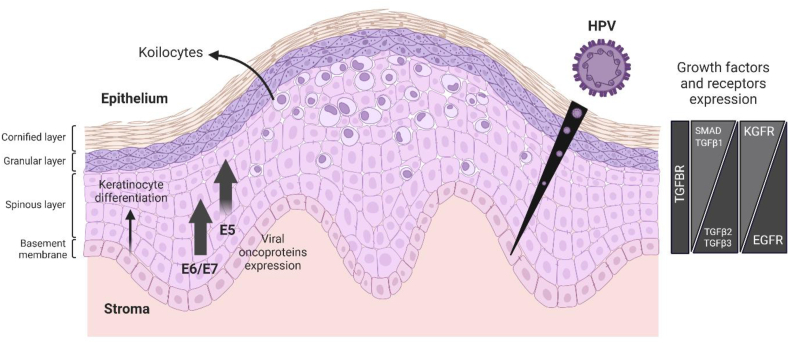
**Expression of host epithelial growth factor receptors (TGFBR/EGFR/KGFR) and viral oncoproteins E5, E6, and E7 in the epithelium during the HPV16 life cycle.** TGFBR exhibits uniform epithelial expression, while TGFβ1 and the SMAD proteins (2–5), the ligand and mediators of TGFβ signaling, respectively, show increased expression in suprabasal cells. In contrast, TGFβ2–3 display lower expression upon epithelial differentiation. EGFR exhibits increased expression in basal cells, and its expression decreased upon the stratification of the epithelium, whereas KGFR shows lower expression in basal cells and increased expression in suprabasal keratinocytes (right). HPV16 initially infects the basement membrane of the epithelium via a microwound that allows its entry into basal cells. Upon keratinocyte differentiation, the virus expresses different oncoproteins (E5, E6, and E7) within the layers of the epithelia, thereby resulting in the formation of koilocytes. HPV expresses the oncoproteins E6/E7 after infection, which have lower expression upon cell stratification, while the oncoprotein E5 exhibit increased expression in differentiating cells where the productive stage of the viral life cycle takes place (left). This figure was made using BioRender (www.biorender.com).

Keratinocyte differentiation is a tightly regulated process essential for maintaining the structure and function of the epidermis [[1], [2], [3]]. This multilayered epithelium is composed of distinct layers: the basal, spinous, granular, and cornified layers, each representing a stage in the maturation of keratinocytes (Fig. 1) [4]. Proliferative basal keratinocytes, anchored to the basement membrane, provide a reservoir of stem and progenitor cells (Fig. 1) [5]. As these cells migrate upwards, they exit the cell cycle and initiate the process of differentiation, characterized by specific changes in gene expression, cytoskeletal organization, and lipid production [2,3]. In the spinous layer, keratinocytes begin to produce structural proteins such as keratins 1 and 10 [3,6]. Further maturation in the granular layer is marked by the formation of keratohyalin granules and the production of filaggrin, crucial for the skin barrier. Finally, in the cornified layer, terminally differentiated keratinocytes, or corneocytes, form a protective barrier, undergoing programmed cell death and desquamation [7].

The differentiation and functional maturation of keratinocytes are closely coordinated with signals from epithelial growth factor receptors (GFRs), which sense and integrate cues from the surrounding epithelial and stromal microenvironment. In normal conditions, GFRs get activated by ligands from the microenvironment and translate these signals into a response in cellular behavior in the epithelium [[8], [9], [10], [11]]. Transforming growth factor-beta receptor (TGFBR), epidermal growth factor receptor (EGFR), and keratinocyte growth factor receptor/fibroblast growth factor receptor 2b (KGFR/FGFR2b) are three GFRs that have significant functions in regulating epithelial homeostasis [[9], [10], [11]], but their expression and their ligands’ expression vary throughout the stratified epithelium, thereby exerting distinctive functions upon cell differentiation. TGFBR can regulate cell growth, proliferation, wound-healing, and epithelial-to-mesenchymal transition (EMT) [10,12]. After the activation of TGFBR, the signal triggers mother against decapentaplegic homolog (SMAD) proteins that induce the transcription of many genes [12,13]. The expression of TGFBR is seemingly uniform in the stratified epithelium, but the expression of its ligand transforming growth factor-beta (TGFβ) 1 increases and TGFβ 2–3 decrease upon cell differentiation [14,15]. Likewise, the SMAD proteins 2–5 exhibit increased expression in suprabasal cells, while the inhibitory SMAD7 shows reduced expression in keratinocytes [[16], [17], [18]].

EGFR, a member of the human epidermal receptor (HER) family, regulates cell proliferation and differentiation during normal conditions and disease [9,19,20]. EGFR or HER1 is found highly expressed at the basal layers of the epithelia, where it regulates differentiation and proliferation of the basal keratinocytes [21,22]. The expression of the receptors HER2 and HER3, however, is upregulated upon differentiation [23,24]. Similarly, KGFR promotes cellular differentiation and proliferation in suprabasal cells, where it is highly expressed because its expression also increases upon the commitment of keratinocytes to differentiation (Fig. 1) [[25], [26], [27]]. The balance between EGFR-driven proliferation and late-stage differentiation, the TGFBR-induced EMT (the opposite process to differentiation), as well as the differentiation-dependent signaling of HER2/HER3 and KGFR, is essential to maintain epithelial homeostasis and ensure the proper turnover and integrity of the epithelial layer [28,29].

TGFBR, EGFR, and KGFR also play distinct yet interconnected roles in regulating immune responses in the epithelium. TGFBR signaling maintains immune tolerance and suppresses excessive inflammation by inhibiting immune cell proliferation and promoting the expression of antimicrobial peptides and the differentiation of Langerhans cells [10,30,31]. By regulating EMT, it influences immune cell recruitment during wound-healing and chronic inflammation [10]. In contrast, EGFR activation promotes epithelial repair and regeneration, indirectly supporting immunity by maintaining barrier integrity [32]. EGFR exhibits both pro-inflammatory and anti-inflammatory functions, recruiting immune cells like neutrophils and macrophages, modulating interactions with the microbiota, and inhibiting the interferon (IFN) signaling [[33], [34], [35]]. Meanwhile, KGFR strengthens barrier function by regulating calprotectin expression and phagocytosis, thereby enhancing innate immune defenses [36,37]. Together, these receptors coordinate epithelial integrity, cytokine signaling, and innate immune responses to maintain epithelial homeostasis and defense against pathogens [38].

Human papillomaviruses (HPVs) can cause malignant lesions in humans due to persistent infections that progress to precancerous lesions and cancer [39,40]. HPVs can persist by evading the immune system and manipulating the epithelium via the expression of viral oncogenes that regulate cellular signaling pathways in keratinocytes and the stromal microenvironment [41,42]. HPV type 16 (HPV16) is a DNA virus that initially infects the basal keratinocytes of the epithelium via microwounds and is maintained in these cells (Fig. 1). Subsequent stages of the life cycle are linked to the stages of keratinocyte differentiation and on the expression of the viral oncoproteins E5, E6, and E7 [41,42]. The oncoproteins E6 and E7 are well-studied proteins that dysregulate the cell cycle in the epithelium; however, there is a recent interest in the oncoprotein E5 (16E5), which can alter cellular proliferation and differentiation, particularly through its interaction with GFRs [[43], [44], [45]].

The functional activities of the oncoprotein 16E5 during epithelial stratification and the HPV differentiation-dependent life cycle are poorly understood. As cells differentiate, the increased expression of E5 suggests its involvement in reprogramming differentiating cells to retain proliferative capacity [43]. 16E5 can also upregulate GFRs, driving cellular growth and proliferation (EGFR), or downregulate GFRs that promote cell proliferation (TGFBR) and early (KGFR) and late differentiation (EGFR) [45]. These contrasting activities seemingly conflict with the need for differentiation to complete the viral life cycle. The assessment of how 16E5 regulates these cellular processes will therefore facilitate understanding how the GFR pathways are implicated in both normal keratinocyte differentiation and HPV16 infection. Furthermore, even though recent findings have described the impact of E5 on GFRs during infection [46,47], an analysis of these cellular pathways in terms of the transition from non-differentiating cells to differentiating cells, and the regulation of this mechanism by 16E5 is still lacking. Accordingly, here we evaluate the regulation of GFRs by 16E5, explore the effect of the oncoprotein over cellular proliferation and differentiation, and assess the regulation of stromal growth factors during the viral life cycle.

## 2 Human papillomavirus 16E5

16E5 is a small hydrophobic protein of 83 amino acids that can be found in the endoplasmic reticulum, the Golgi apparatus, and the nuclear membrane of infected cells [45,48,49]. 16E5 is a pore-forming protein with the ability to fuse and modify host cellular membranes [[51], [52], [53]], a function that likely depends on its expression levels during the viral life cycle. Moreover, mutations in the HPV genome can influence viral persistence, immune evasion, and oncogenic potential [54]. In the E5 gene, specific mutations may enhance its stability and function, leading to increased cell proliferation, immune suppression, amplification of viral DNA, and activation of oncogenic pathways [[55], [56], [57]].

16E5 regulates many cellular activities during infection, including cellular transformation [41,45,51]. This oncoprotein can particularly promote immune evasion during the viral life cycle by impairing the immune response through the alteration of natural killer (NK) cell response, TGFβ signaling, and IFN signaling pathways [33,58]. Characteristically, type I and III IFNs activate the JAK-STAT pathway to induce antiviral interferon-stimulated genes (ISGs) [59], but 16E5 suppresses this pathway by inhibiting the TGFβ signaling and promoting EGFR signaling, thereby reducing ISG expression [33]. 16E5 also downregulates the STING pathway [60], decreasing IFN production in infected keratinocytes. Additionally, E5 disrupts stromal IFN responses by impairing crosstalk between infected epithelial cells and fibroblasts, creating an immunosuppressive environment [58]. By also downregulating MHC class I, E5 impairs antigen presentation, while E6 and E7 further inhibit the IFN signaling by degrading p53, disrupting Rb, and suppressing STAT1, CDK8, and IRF3 [[60], [61], [62], [63]]. The combined activities of E5, E6, and E7 result in a synergistic disruption of cellular homeostasis, significantly contributing to oncogenesis. These mechanisms enable persistent infection and drive HPV-related disease progression.

**Fig. 2:**
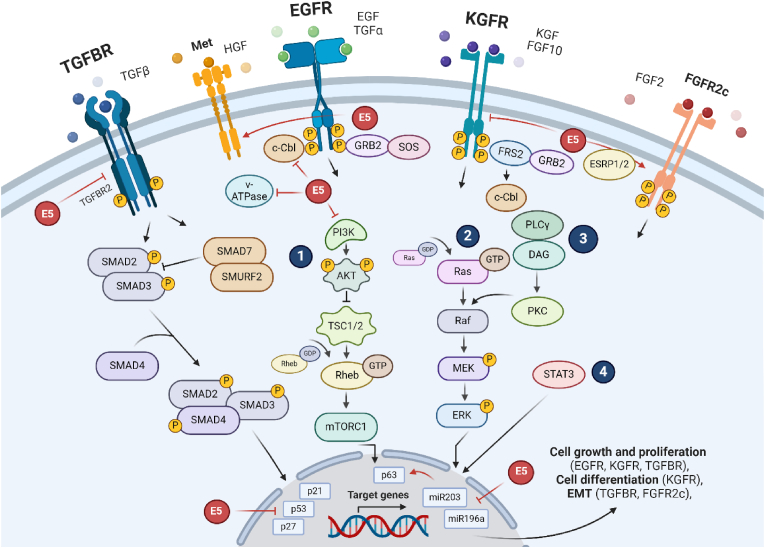
**Epithelial growth factor receptors (TGFBR/EGFR/KGFR) cellular signaling and their regulation by 16E5.** TGFBR canonical signaling starts with the activation of TGFBR2 by ligand binding (TGFβ). Next, the receptor recruits TGFBR1, and once activated it recruits the SMAD proteins 2/3. These proteins then recruit SMAD4 and form the SMAD protein complex (SMAD2/3/4) that initiates nuclear translocation. In turn, SMAD7/SMURF2 downregulate this signaling by the degradation of the SMAD2/3 complex. Likewise, TGFBR non-canonical signaling includes the activation of PI3K/AKT (**1**) and MAPK (**2**) pathways. EGFR/KGFR signalings start with their activation by ligand binding, for instance, EGF and KGF, respectively. Next, they can activate several pathways such as PI3K/AKT (**1**), MAPK (**2**), PLCγ/PKC (**3**), or STAT3 (**4**). These overall pathways result in the stimulation of their target genes in the nucleus that leads to the regulation of cell growth and proliferation (EGFR, TGFBR), cell differentiation (EGFR, KGFR), and EMT (TGFBR, FGFR2c). During the HPV life cycle, 16E5 downregulates TGFBR2, and therefore, the TGFβ signaling pathway. The oncoprotein may also upregulate EGFR by inhibiting both c-Cbl and v-ATPase, and therefore, preventing its degradation and endocytic traffic, respectively. This EGFR enhancement by 16E5 might result in the upregulation of Met, another growth factor receptor, and the inhibition of p27. Alternatively, 16E5 may downregulate KGFR and promote the expression of the variant FGFR2c as a result of FGFR2 splicing by ESRP1/2. 16E5 might also inhibit this receptor by downregulating PI3K/AKT pathways and upregulating p63; p63 upregulation might also be accomplished by the 16E5 inhibition of miR-203. In addition, 16E5 might downregulate p53 and p21. 16E5 might also downregulate miR-196a, leading to cell growth and proliferation.

During the viral life cycle, 16E5 is expressed from polycistronic mRNAs originating at the E6 open-reading frame (ORF) in undifferentiated basal keratinocytes, making it the fourth ORF, which likely reduces its translation efficiency in these cells [43]. As keratinocytes undergo differentiation, there is a shift in viral transcriptional activity. Late-stage transcripts, abundantly produced in differentiated epithelial cells, position E5 as the second ORF, thereby potentially enhancing its expression in suprabasal cells [41]. This particular viral expression facilitates the induction and regulation of important cellular processes in the epithelium. For instance, E5 suppresses the expression of cyclin-dependent kinase inhibitors such as p21 and p27 to promote cell cycle progression and impairs autophagy by inhibiting the tumor suppressor protein p53 (Fig. 2) [45,64]. Upon epithelial stratification, 16E5 suppresses early differentiation and along with E6 promotes koilocytosis, which is the vacuolization of cells that develop irregular nucleus and cytoplasm (Fig. 1) [44,45,65,66]. Nevertheless, 16E5 was also recently found to promote differentiation of HPV-containing cells in the granular layer of the epithelium [46]. This distinct regulation of proliferation and differentiation by 16E5 and its increased expression in the mid-layers of the epithelium during the viral life cycle demonstrate its dual functional activities. Additionally, due to fact that the GFR pathways are involved in the regulation of these processes upon ligand-binding, 16E5 might control the expression of their ligands in the epithelium and the stroma. However, the role of 16E5 in the regulation of the growth factors signaling in the microenvironment need further assessment [46].

## 3 Regulation of TGFBR signaling by E5

The TGFβ canonical signaling pathway gets triggered with the binding of the ligands (TGFβ1, 2, or 3) to the receptors (TGFBR1, 2, or 3), usually TGFBR2, which recruits TGFBR1. Next, the activated TGFBR1 phosphorylates SMAD2/3, which leads to the recruitment of SMAD4. Then the complex SMAD2/3/4 translocates to the nucleus, promoting the transcription of the target genes. The activation of the receptor can also trigger the TGFβ non-canonical signaling that includes the induction of PI3K/AKT and MAPK pathways (Fig. 2) [10,12,13]. In normal conditions, TGFβ1 suppresses the growth of keratinocytes by inhibiting c-myc, a protein that can activate the transcription of many pro-proliferative genes. This way, the TGFβ pathway downregulates cellular proliferation [67,68]. TGFβ upregulates TGFBR2 by increasing its levels at the cell surface [69].

The cell growth inhibition induced by the TGFβ signaling is disturbed by HPV during its life cycle. The expression of the oncoproteins E6 and E7 might suppress TGFβ2 via the downregulation of p53 and pRb, respectively [70,71]. E7 prevents TGFβ signaling, which in turn decreases E7 expression by blocking the binding of NF1/Ski complexes with the HPV16 upstream regulatory region [71,72]. Moreover, the virus also exerts an effect on the expression of the receptors. The virus was found to downmodulate TGFBR1 and TGFBR2 via E6 and E7 [[72], [73], [74], [75]]. Intriguingly, several variants of both TGFβ1 and TGFBR1 have been recently associated with the survival of HPV-induced cancer patients [[76], [77], [78]]. Thus, the oncoproteins E6/E7 employ several mechanisms that lead to TGFβ suppression, thereby inhibiting TGFβ functional activity in keratinocytes [79,80]. Nevertheless, during HPV16 infection, TGFβ seemingly suppresses the expression of cyclins A1, A2, B1, and B2 and upregulates cyclin-dependent kinase inhibitors p21, p57, and p15, which is mediated by the action of SMAD [81]. This event might occur during early stages of the viral cycle [81], suggesting that TGFβ signaling is still able to induce cell growth inhibition at the beginning of the infection.

The role of 16E5 in the regulation of TGFβ has been recently described. By assessing low-grade and high-grade intraepithelial cervical lesions (LSIL, HSIL), French et al. demonstrated that 16E5 downregulates TGFBR2 [82]. Further analyses showed that this suppression results in the attenuation of SMAD4 nuclear translocation, and therefore, in the downregulation of the TGFβ1 signaling pathway [82]. Similarly, a recent study by Woodby et al. described that TGFβ1 transcripts were reduced in HPV16-containing cells [71]. Interestingly, TGFβ2 transcripts were increased, which suggested that the virus regulates TGFβ isoforms distinctively. The authors determined that TGFβ1 inhibits HPV transcripts in undifferentiated (monolayer) and differentiated conditions (methylcellulose), in a differentiation-independent manner [71]. Similarly, TGFβ can increase the IFN response during HPV infection and reverse the methylation of the IFN-κ promoter, which is first induced by the virus [71]. Therefore, HPV16 may downregulate TGFβ1 signaling to promote viral replication and immune evasion. Intriguingly, the downregulation of this TGFβ isoform may result in the upregulation of the TGFβ2 signaling, leading to increased viral oncoproteins production (E6/E7) or a yet undescribed viral interaction with the microenvironment [71]. Additionally, TGFBR2 transcripts and proteins levels have also been detected in primary human foreskin keratinocytes (HFKs) vs. HPV16-containing cells [83], and 16E5 was found to downregulate the TGFβ signaling via the suppression of TGFBR2 [33]. The authors demonstrated that the TGFβ signaling activates the IFN-κ immune response and that cells harboring mutant HPV16 genomes that could not express E5 (E5 Stop cells) exhibit increased levels of SMAD3 and TGFBR2, in comparison to HPV16-containing cells [33]. 16E5 is therefore responsible for the downregulation of TGFβ1, upregulation of TGFβ2, the reduction of TGFBR2, and the subsequent immune evasion strategies in the epithelium [33,71].

## 4 Regulation of EGFR signaling by E5

EGFR can bind to different ligands from the microenvironment, which include the epidermal growth factor (EGF), transforming growth factor-alpha (TGFα), and the heparin-binding EGF (HBEGF) [32,84,85]. The subsequent activation by ligand-binding triggers different cellular signals to phospholipase C-γ (PLCγ), protein kinase C (PKC), MAPK, PI3K/AKT, Src, and STAT3 pathways (Fig. 2) [25,84,[86], [87], [88], [89], [90]]. MAPK/Src pathways are involved in cell proliferation, while PI3K/AKT in cell growth, and STAT3 in cell migration and differentiation [84,90]. Upon ligand-binding, the receptor can get internalized and either recycled or degraded in the cell.

The relationship between EGFR and HPV begins upon viral entry into the host cells. Mikuličić et al. described that the activation of the extracellular signal-regulated kinases (ERK1/2) pathway, via the shedding of EGFR, triggers the formation of an endocytic entry platform that facilitates HPV16 infection [91]. Furthermore, Rosenberger et al. demonstrated that the EGFR signaling could regulate the splicing of E6/E7 in the stratified epithelium, which works as an additional mechanism used by HPV to complete its life cycle [92]. Akerman et al. also described that the E6/E7 proteins increase the levels of EGFR [93], while other studies found that the β-catenin and JNK signaling pathways were regulated via the activation of EGFR by E6 [94].

Regarding the role of 16E5, the oncoprotein might activate EGFR in a ligand-dependent manner [46,47]. Tomakidi et al. found that 16E5 induces the overexpression of EGFR in keratinocytes raft cultures in all the layers of the epithelia, but particularly in the basal layer [95]. Similarly, 16E5, expressed from the context of the complete HPV genome, was found to upregulate EGFR in monolayers of HFKs, and upon ligand-binding of EGF, EGFR upregulates Met, another GFR (Fig. 2) [83]. Further studies confirmed the HPV-induced upregulation of EGFR [33]. In these experiments, EGFR levels were decreased in cells containing an HPV16 genome unable to express E5, in comparison with the levels observed in cells containing wild type HPV16 [33]. Overall, there is consensus on the role of 16E5 in enhancing EGFR expression, although the mechanisms are unclear [33,46,96]. E5 may decrease the degradation of EGFR by inhibiting its interaction with c-Cbl and avoid its endocytic trafficking by suppressing the vacuolar ATPase (v-ATPase) of the receptor, thereby upregulating the EGFR signaling (Fig. 2) [44,45,97]. Likewise, 16E5 was found to promote the recycling of EGFR and cell cycle progression via the activation of EGFR and the inhibition of p27^Kip1^ (Fig. 2) [45,98].

The function of E5 in suprabasal keratinocytes has not received much attention, even though E5 levels are highest in differentiating cells [41,99]. The lower expression of EGFR in suprabasal cells after keratinocyte differentiation may suggest that the regulation of EGFR by E5 is distinct in these differentiating cells [46]. In fact, Trammel et al. observed an 16E5-dependent increase in both the levels and activation of EGFR in differentiated granular layer cells [46]. By stimulating EGFR in cells committed to differentiation, the authors found increased cell differentiation and enhanced late viral gene expression [46]. The authors suggested a model by which 16E5 promotes the EGFR signaling in both basal and granular layers to inhibit the IFN pathway and promote cell differentiation, respectively [46]. Nonetheless, neither 31E5 nor 18E5 regulate EGFR in basal cells, and instead, they seemingly exhibit a differentiation-dependent function in the upper layers of the epithelium [99,100]. These contradicting results of E5 regulation over EGFR in undifferentiated cells may be attributed to the use of different cell lines, HPV genotypes, and therefore distinct E5 proteins [83,99,100]. Thus, there is still a need for clarifying the role of 16E5 on EGFR in the epithelium. Since EGFR signaling can activate multiple intracellular pathways, further analyses are needed to elucidate the signals that lead to proliferation and differentiation.

## 5 Regulation of KGFR signaling by E5

KGFR or FGFR2b expression can be regulated by p63, a protein that induces paracrine signaling to the stroma from the epithelium, promoting the expression of stromal ligands [25,101,102]. KGFR might regulate early differentiation of keratinocytes through the PI3K/AKT signaling pathways and get triggered by binding of stromal ligands that lead to cell differentiation and proliferation (Fig. 2) [26,103]. Following its activation, KGF or fibroblast growth factor 7 (FGF7) drives the degradation of the receptor by inducing FRS2‐mediated recruitment of c‐Cbl, while FGF10 promotes its recycling [[104], [105], [106]]. KGF is known to promote early differentiation and control autophagy of epithelial cells, and it has also been involved in promoting cell migration via the activation of NF-κB [107]. FGF10 is also a potent ligand that can induce early differentiation markers such as K1 and K10 and promote the late differentiation marker filaggrin in response to Ca2+ signal [108].

The fibroblast growth factor receptors (FGFR) 1–3 can all be spliced to an IIIb isoform (“epithelial”) and an IIIc isoform (“mesenchymal”) by the Epithelial Splicing Regulatory Proteins 1 and 2 (ESRP1/2) [109,110]. The FGFR2 gene can be spliced to KGFR/FGFR2b or the variant FGFR2IIIc/FGFR2c [101]. The expression of FGFR1c (spliced variant of FGFR1) is associated with EMT during wound-healing, where TGFβ promotes a switching expression from KGFR to FGFR1c [111]. In contrast, the expression of FGFR2c is increased during carcinogenesis, including HPV-induced cancer [[112], [113], [114]]. Intriguingly, FGFR2c leads to the pathological type III EMT (loss of keratins/vimentin expression and related to cancer progression) and not the type II EMT (loss of cell adhesion with the expression of keratins) usually observed in KGFR/FGFR1c-mediated wound-healing. The activation of these IIIc variant receptors results in the reorganization of cellular shape and actin, complemented by the control of EMT markers via the regulation of transcription factors and miRNAs [115]. Therefore, KGFR/FGFR2b is the spliced receptor that should be at higher levels in normal keratinocytes, while the presence of the variants FGFR1c/FGFR2c makes the cells acquire a more mesenchymal phenotype related to an ongoing EMT process. The activation of EMT by FGFR2c was further studied by Ranieri et al., who observed that the signaling of these receptors triggers PKCε [113]. PKCε mediates the expression of EMT markers via the induction of STAT3, FRA1, and SNAIL1. The authors confirmed these results by inactivating ESRP1 [113].

HPV16 might affect this GFR via several mechanisms. The oncoproteins E6 and E7 may downregulate stromal AKT to enhance stromal KGF and promote invasion of keratinocytes through KGFR [116]. This was demonstrated by Cichon et al., who employed primary human foreskin fibroblasts (HFFs) and HFKs, where the knockdown of stromal AKT isoforms showed that active AKT2 is responsible for the invasion of keratinocytes expressing E6/E7 viral proteins [117,118]. In addition, they reported that stromal interleukin 1 beta (IL1β) inhibits stromal KGF in response to AKT2 depletion, which leads to the downregulation of KGFR expression in the epithelium [118]. Moreover, 16E5 might downregulate KGFR to induce FGFR2c expression via splicing, thereby promoting EMT in the epithelium (Fig. 2) [65,102,119]. By using a human keratinocyte cell line (HaCaT), Belleudi et al. demonstrated that 16E5 could inhibit autophagy by impairing p53 function, which forces the expression and activation of KGFR [114,120]. The research team suggested that the suppression of autophagy might be a mechanism by which 16E5 delays epithelial differentiation to promote viral amplification in differentiating cells [114]. Similarly, Purpura et al. reported that 16E5 can alter keratinocyte differentiation through the downmodulation of KGFR/PI3K/AKT signaling and regulation of p63, thereby decreasing K1 expression (Fig. 2) [65,114]. A reduction of FGFR2 transcripts has also been observed in HPV16-containing cells, further confirming the viral downregulation of this receptor [83]. Interestingly, Ren et al. described that episomal E2/E4/E5 can drive an alternative carcinogenic pathway that activates an FGFR network signature in HPV-positive cancers, which can be hindered via FGFR and mTOR inhibitors [40,121].

## 6 Switching regulation from EGFR to KGFR by HPV16

The differences between EGFR and KGFR in cellular differentiation were first described by Marchese et al., who reported that the expression of terminal differentiation markers such as K1 and filaggrin were associated to KGF and that EGF/TGFα blocked or delayed their appearance, suggesting different roles of the receptors during keratinocyte differentiation [122], an observation that may differ in models with complete cell differentiation. Nonetheless, due to the fact that EGFR and KGFR exhibit increased expression in basal and suprabasal cells, respectively, and both receptors are regulated by 16E5, there might be a switch regulation of GFRs by the oncoprotein [21,46,99,113].

Initially, Barbaresi et al. showed that 16E5 dysregulates cellular differentiation in HaCaT human keratinocyte cultures [123]. By using the same cell model, Belleudi et al. found that 16E5 alters the endocytic trafficking and expression of KGFR in cells committed to differentiation [124]. Consequently, the authors suggested that 16E5 may induce EGFR signaling in basal layers, while in suprabasal cells, it decreases KGFR signaling [124]. Further observations were described by Wasson et al. using HPV18 and organotypic raft cultures from HFKs [99]. Their research demonstrated that 18E5 promotes cell cycle progression in suprabasal layers of the epithelium [99,100]. The observed enhancement of EGFR by 18E5 was focused on suprabasal keratinocytes and the authors reported that 18E5 was not responsible for the EGFR upregulation in the basal layer [99]. These findings are in contrast with previous reports where the regulation of EGFR by 16E5 is described in the basal cells but agrees with a report studying 31E5, and Genther et al. who described the role of 16E5 specifically in the productive stage of the viral life cycle [55].

Wasson et al. also reported that EGFR is needed for the expression of cyclin B in differentiating keratinocytes and that the expression of EGFR leads to the downregulation of KGFR [99]. Finally, they observed the downregulation of KGFR by 18E5 and an unknown role of the AKT signaling in the reduction of differentiation by the oncoprotein [99]. Although Wasson et al. demonstrated significant activities of 18E5 in the viral life cycle during keratinocyte differentiation, there is still the question if these results are similar in the other high-risk HPV genotypes and different cell models [99]. Altogether, these findings suggest that there is a switching regulation of EGFR from the basal layer, which may be attributed to E6/E7 oncoproteins, to the suprabasal layers, where EGFR and KGFR functions are primarily regulated by E5 [44,45,94,125].

## 7 16E5 effects on cellular proliferation and keratinocyte differentiation

The role of 16E5 in enhancing EGFR is well-supported by various studies, though conflicting evidence exists [33,46,47,83,96,97]. Some studies suggest that E5 does not regulate EGFR or that its enhancement is mediated by E6 and E7 oncoproteins [93,94,99,100,126]. Differences in experimental models, including HFKs, HFFs, cancer cell lines, immortalized keratinocytes (HaCaT), and organotypic raft cultures, may explain these discrepancies. Notably, a 16E5-EGFR relationship is consistently observed in organotypic raft cultures of HFKs/HFFs, highlighting the potential of these models to clarify 16E5's role in EGFR upregulation during the viral life cycle [33,99]. This EGFR upregulation, particularly in basal keratinocytes, likely contributes to HPV's immune evasion mechanisms [33,46,83].

16E5 also influences cellular proliferation and differentiation by downregulating TGFBR2 and miR-196a [127], promoting growth and suppressing apoptosis. Furthermore, 16E5 suppresses KGFR and miR-203 in differentiating cells, enhancing p63 expression, which is crucial for late viral expression and genome amplification in keratinocytes [65]. However, this suppression conflicts with HPV's dependence on keratinocyte differentiation, raising questions about 16E5's regulatory mechanisms. Studies using HaCaT cultures have provided key insights [65,124], but further research across diverse models is needed to confirm 16E5's effects on KGFR and epithelial differentiation, and to fully understand its role in creating a hyperproliferative epithelial state in the basement membrane [47].

## 8 HPV16 regulation of stromal growth factors

**Fig. 3:**
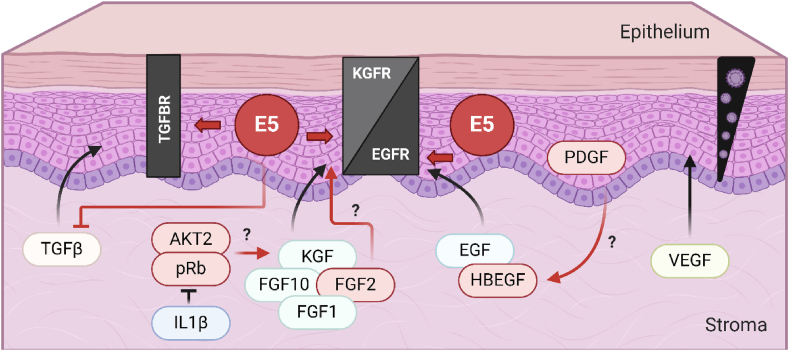
**Stromal growth factors signaling and the interplay of TGFBR/EGFR/KGFR with the viral oncoprotein 16E5 in the epithelium**. TGFBR, particularly TGFBR2, is downregulated by 16E5 in epithelial cells. The oncoprotein might suppress stromal TGFβ, which can bind to epithelial TGFBR. EGFR increased expression in basal cells may be enhanced by the oncoprotein 16E5, promoting cellular proliferation. This receptor can bind to several ligands such as EGF and HBEGF, which can be derived from the epithelium or the stroma. KGFR exhibits increased expression in suprabasal cells, where it gets downregulated by 16E5. KGFR can bind to several ligands such as KGF/FGF7, FGF10, FGF1, while FGF2 is specific for the variant FGFR2c, which might be expressed as the result of E5-induced splicing. Additional growth factors might move from stromal cells to the epithelium such as VEGF, which might be activated by the oncoprotein E6. HBEGF might be induced by epithelial PDGF during HPV-induced cancer. Additionally, the viral disruption of stromal pRb/AKT2 may alter stromal KGF signaling, and therefore epithelial KGFR expression, a mechanism that can be suppressed by the production of stromal IL1β. This figure was made using BioRender (www.biorender.com).

The stromal microenvironment plays a critical role in the HPV16 life cycle by influencing stromal growth factors and cytokines to promote angiogenesis, immune evasion, and keratinocyte invasion [58,117,[128], [129], [130], [131]]. HPV16 can regulate stromal growth factors such as FGF1, FGF2, and TGFβ, which interact with epithelial receptors like TGFBR, EGFR, and KGFR [117,121,132]. For instance, studies in transgenic mice and organotypic raft cultures have shown that HPV16 upregulates stromal FGF1 and downregulates TGFβ signaling, mediated by E5 (Fig. 3) [45,82]. This downregulation involves decreased expression of stromal SMAD3 and TGFBR3, with the inhibition reversed in the absence of E5. Additionally, FGF2 triggers EMT in cervical epithelial cells expressing E5, highlighting the virus's role in modulating the stromal environment to favor viral persistence and progression [45,65,116,119,133].

HPV16 also manipulates stromal-epithelial interactions to enhance cellular invasion. Loss of pRb in stromal cells upregulates KGF, promoting KGFR expression in epithelial cells and facilitating invasion (Fig. 3) [134]. This effect is mediated through stromal AKT2 signaling and is amplified in keratinocytes expressing E6 and E7 oncoproteins [118]. Other stromal factors, such as HBEGF, platelet-derived growth factor (PDGF), and vascular endothelial growth factor (VEGF), are implicated in epithelial proliferation and cancer progression [50,135,136]. VEGF, activated by E6, enhances EGFR expression, while VEGFR expression may be induced by E5 through EGFR activation (Fig. 3) [[137], [138], [139]]. Furthermore, HPV16 suppresses ISGs and extracellular matrix remodeling genes in stromal fibroblasts through E5, disrupting stromal innate immunity and epithelial-stromal crosstalk [58]. This suppression increases late keratinocyte differentiation and viral late gene expression, demonstrating a paracrine role for stromal IFN signaling in controlling the HPV16 life cycle [58]. These findings underscore the intricate regulation of stromal factors and their receptors by HPV16, highlighting their importance in viral pathogenesis and HPV-associated cancers.

## 9 Conclusions and future directions

Here we highlight the intricate interplay between 16E5 and GFRs such as TGFBR, EGFR, and KGFR during the viral differentiation-dependent life cycle. 16E5 modulates TGFBR and EGFR through opposing mechanisms, downregulating TGFβ signaling while promoting EGFR recycling and suppressing its degradation. 16E5's effect on EGFR in differentiating cells promotes viral replication and may be synergistically enhanced by the oncoproteins E6 and E7. In contrast, 16E5 downregulates KGFR by inhibiting PI3K/AKT signaling, inducing the FGFR2c splice variant, or increasing EGFR levels. This paradoxical inhibition of KGFR, critical for early keratinocyte differentiation, may represent a stage-specific regulatory switch where E5 promotes hyperproliferation in the basal layer and viral replication in the suprabasal layer. Nonetheless, further studies investigating this potential 16E5-induced hyperproliferative state in the basal cells are required. Additionally, 16E5's potential role in modulating EMT through TGFβ/KGFR interactions and p63 regulation underscores its complexity, while the influence of stromal immune signaling on late viral genes, differentiation, and KGFR expression necessitates further exploration using models that include complete cell differentiation [70,[140], [141], [142], [143]].

Future studies should investigate how HPV16 regulates additional GFRs such as Met, VEGFR, IGF1R, Notch, and other members of the FGFR and HER families, particularly via E5's functional activities [[144], [145], [146]]. The differential expression of ligands like FGF22 in basal and suprabasal epithelial layers [111,147] might suggest distinct roles in viral replication and differentiation. Regarding the 16E5-induced expression of the harmful spliced variant FGFR2c, further development of protein isoform-centric therapeutics [148], such as splicing modulators [[149], [150], [151]], oligomeric assemblies [152], antibodies [101], and inhibitors [101,121,153], remains essential for their potential application in HPV-driven cancer patients. Unraveling the virus's ability to manipulate epithelial GFRs and immune responses could clarify mechanisms underlying hyperproliferation, immune evasion, and persistence in the basal layer, as well as the implications for carcinogenesis. Understanding these processes will provide critical insights into HPV16's life cycle and its progression to malignancy, paving the way for targeted therapeutic interventions.

## CRediT authorship contribution statement

**Mariano A. Molina:** Writing – review & editing, Writing – original draft, Conceptualization. **Sneha Biswas:** Writing – review & editing. **Omar Jiménez-Vázquez:** Writing – review & editing. **Jason M. Bodily:** Writing – review & editing, Writing – original draft, Conceptualization.

## Funding

This research did not receive any specific grant from funding agencies in the public, commercial, or not-for-profit sectors.

## Declaration of competing interest

The authors declare that they have no known competing financial interests or personal relationships that could have appeared to influence the work reported in this paper.

## Data Availability

No data was used for the research described in the article.
